# A bifactor model of the Posttraumatic Growth Inventory

**DOI:** 10.1080/21642850.2014.905208

**Published:** 2014-04-23

**Authors:** Barna Konkolÿ Thege, Éva Kovács, Piroska Balog

**Affiliations:** ^a^ Department of Psychology, University of Calgary, 2500 University Drive NW, Calgary, CanadaT2N 1N4; ^b^ Institute of Behavioral Sciences, Semmelweis University, Budapest, Hungary; ^c^ Department of Social Work, John Wesley Theological College, Budapest, Hungary

**Keywords:** Posttraumatic Growth Inventory, psychometric properties, factor structure, confirmatory factor analysis, bifactor model

## Abstract

*Purpose*: The Posttraumatic Growth Inventory (PTGI) is a self-administered measurement instrument designed to provide information concerning positive psychological changes after a traumatic life event. The aim of the present study was to examine the psychometric properties of the PTGI in a Hungarian sample. By examining a bifactor model of the instrument, we also wanted to contribute to the establishment of an evidence-based practice concerning the use of different score types (total score versus subscale scores). *Methods*: Altogether, 691 Hungarian respondents (82.2% female; *M*_age_ = 33.0 ± 13.4 years), who experienced some kind of trauma or loss, participated in this study. *Results*: A series of confirmatory factor analyses revealed that among the tested first- and second-order models, a bifactor model provided the best-fit to our data (*χ* ^2^/df = 4.32, Comparative Fit Index = .91, root mean square error of approximation = .07, standardized root mean square residual = .04). Further, the Hungarian version of the PTGI showed high internal consistency (Cronbach's alpha = .93, omega total = .95, omega hierarchical = .87) and test–retest reliability (*r* = .90; *p* < .01) coefficients. However, omega hierarchical coefficients (.14–.40) and explained variance values (.05–.10) for the subscales were low. *Conclusions*: The present study provided empirical support for the psychometric adequacy of the Hungarian adaptation of the PTGI and suggests that only the total and not the subscale scores of the inventory should be used.

## 1. Introduction

The Janus-faced phenomenon of losses involves suffering and pain on the one hand and positive changes through adjustment to trauma on the other (Maercker & Zoellner, 2004). The experience of these latter positive changes as a result of highly challenging life crises has been labeled *posttraumatic growth* by Tedeschi and Calhoun (1996). In the last decade and a half, many investigations have examined posttraumatic growth in different groups of trauma survivors including the bereaved (Engelkemeyer & Marwit, 2008), war veterans (Feder et al., 2008; Kaler, Erbes, Tedeschi, Arbisi, & Polusny, 2011), political prisoners (Salo, Punamaki, Qouta, & El Sarraj, 2008), victims of traffic accidents (Nishi, Matsuoka, & Kim, 2010), survivors of terrorist attacks (Posta, 2010), earthquake survivors (Gao et al., 2010), people infected with HIV (Nightingale, Sher, & Hansen, 2010), patients suffering from rheumatoid arthritis (Dirik & Karanci, 2008), cancer patients (Brunet, McDonough, Hadd, Crocker, & Sabiston, 2010; Zwahlen, Hagenbuch, Carley, Jenewein, & Buchi, 2010), and individuals suffering other losses including divorce (Lamela, Figueiredo, Bastos, & Martins, 2014) or exile (Teodorescu et al., 2012).

Several instruments were developed for the assessment of the subjective experience of positive psychological changes after traumatic events. One of the most commonly used is the Posttraumatic Growth Inventory (PTGI) developed by Tedeschi and Calhoun (1996). Beyond the original English-language version of the scale, it is now widely available in many other languages – including Bosnian (Powell, Rosner, Butollo, Tedeschi, & Calhoun, 2003), Chinese (Ho, Chan, & Ho, 2004), Dutch (Jaarsma, Pool, Sanderman, & Ranchor, 2006), German (Maercker & Langner, 2001), Hebrew (Lev-Wiesel & Amir, 2003), Italian (Prati & Pietrantoni, 2014), Japanese (Taku et al., 2007), Portuguese (Lamela et al., 2014; Teixeira & Pereira, 2013), Spanish (Weiss & Berger, 2006), and Turkish (Dirik & Karanci, 2008). Besides the cultural adaptations of the instrument, several researchers have developed shortened (Cann et al., 2010), revised (Kilmer et al., 2009; Lau et al., in press), or altered versions of the questionnaire for special populations as children or adolescents (Cryder, Kilmer, Tedeschi, & Calhoun, 2006; Taku, Kilmer, Cann, Tedeschi, & Calhoun, 2012).

Many studies have also investigated the psychometric properties of the PTGI. However, ambiguous results emerged in the different studies leaving an important question unanswered: whether posttraumatic growth is a single unidimensional construct or a collection of different components. For example, the principal component analysis performed on the 21-item questionnaire by the test developers revealed five components which explained 62% of the total variance (Tedeschi & Calhoun, 1996). These components were labeled as Relating to Others (seven items), New Possibilities (five items), Personal Strength (four items), Spiritual Change (two items), and Appreciation of Life (three items).

However, in other samples of subsequent investigations, one- (Joseph, Linley, & Harris, 2005; Polatinsky & Esprey, 2000; Sheikh & Marotta, 2005), two- (Sheikh & Marotta, 2005), three- (Joseph et al., 2005; Powell et al., 2003; Weiss & Berger, 2006), four- (Ho et al., 2004; Maercker & Langner, 2001; Taku et al., 2007), and five-factor (Anderson & Lopez-Baez, 2008; Jaarsma et al., 2006; Linley, Andrews, & Joseph, 2007; Morris, Shakespeare-Finch, Rieck, & Newbery, 2005; Teixeira & Pereira, 2013) solutions have been identified. When conducting confirmatory factor analyses, the five-factor model of the PTGI showed the best-fit compared to other models tested in the same samples – supporting the original conception by Tedeschi and Calhoun (Brunet et al., 2010; Hooper, Marotta, & Depuy, 2009; Linely et al., 2007; Prati & Pietrantoni, 2014; Taku, Cann, Calhoun, & Tedeschi, 2008).

Because of the theoretically plausible and consistently found correlation among the five factors of the construct, the possible existence of an underlying general posttraumatic growth second-order factor has emerged (Joseph et al., 2005; Sheikh & Marotta, 2005) that can generally assess the positive changes after a traumatic event. This concept led to a heterogeneous research practice where some researchers used only the total score of the PTGI to measure posttraumatic growth (Osei-Bonsu, Weaver, Eisen, & Van der Wal, 2012; Scrignaro, Barni, Bonetti, & Magrin, 2011; Widows, Jacobsen, Booth-Jones, & Fields, 2005), while other researchers also used or preferred the subscale scores of the instrument (Morris et al., 2005; Taku et al., 2008). However, the decision for whether the components of a construct can be analyzed apart from the main construct or not should be based on solid confirmatory factor analytic research evidence which – compared to the amount of published literature using this instrument – might be considered scarce for the PTGI.

**Figure 1.**
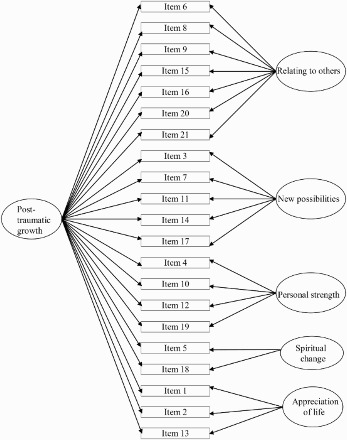
A bifactor model of the PTGI.

A possible way to clarify the relationships between the components and the main construct itself is to conduct a confirmatory factor analysis using a bifactor model (Gibbons, Immekus, Bock, & Gibbons, 2007). Similar to conventional second-order models, the bifactor model enables the estimation of specific and general factors simultaneously (Mészáros, Ádám, Szabó, Szigeti, & Urbán, 2014). However, this kind of model applies a latent structure where all items load onto a general dimension and onto one of several specific factors at the same time (Figure 1) – considered a more realistic representation of a complex psychological construct (Reise, Scheines, Widaman, & Haviland, 2013). Contrary to the traditional first- and second-order models, the general and all the specific factors are uncorrelated in a bifactor model. Consequently, the general factor reflects what is common among the items and represents the individual differences of the target dimension, while the specific factors represent item response variance not accounted for by the general factor (Reise, Moore, & Haviland, 2010). In this way, a bifactor model helps to determine whether domain-specific factors can be used in a meaningful way over and above the general factor, for instance, when predicting external variables.

Therefore, the main goal of our study was to test alternative confirmatory factor analytic models of posttraumatic growth – including bifactorial solutions – to better understand the dimensionality of the construct measured by the PTGI. Second, we intended to present data on the psychometric properties of the Hungarian adaptation of the instrument.

## 2. Methods

### 2.1. Sample and procedure

Our convenience sample consisted of 691 respondents, 310 (50.1%) of whom answered the questionnaire in a paper–pencil format while the remaining answered electronically. The data were collected in Hungary between 2009 and 2012; participants were recruited using electronic mailing lists and social networking websites. Participation in the study was completely voluntary and anonymous. Seventy-two individuals (10.4%) of the sample did not complete the whole questionnaire and their data were excluded from the analyses. In order to investigate the temporal stability of the instrument, 35 individuals were selected randomly from the original sample to complete the questionnaire again six weeks after the first administration. Of these, 31 questionnaires were returned (88.6% response rate).

Our sample consisted of 509 women (82.2%) and 110 men (17.8%). The age range was 18–82 years and the mean age was 33 years (SD = 13.4). Level of educational attainment in the sample was as follows: 15 participants (2.42%) had elementary level education or less, 18 (2.91%) finished secondary vocational school, 414 (66.88%) finished secondary grammar school, and 110 (17.77%) graduated from college/university.

Two additional questions (see later in Section 2.2) administered before the standard items of the PTGI made it possible to gain information about the adverse/traumatic event the respondents experienced and the date it happened. Almost a third (29.1%) of the sample reported the death of a related person, 27.1% reported the break-up of an intimate relationship or divorce, 12.8% reported job loss, 8.9% mentioned the illness of a significant person, 8.9% indicated their own illness, 6.6% indicated family problems, 1.3% mentioned injury-producing accidents, 1.3% cited loss of home, and 4% reported a variety of other negative life events (e.g. being abused, being a victim of rape or violent robbery). Two hundred and twenty-two (35.86%) participants reported these events occurred less than 12 months before data collection, 110 (17.70%) between 13 and 24 months, 263 (42.49%) between 25 and 60 months, and 24 (3.88%) between 61 and 120 months.

### 2.2. Instruments

In addition to basic socio-demographic characteristics, the PTGI (Tedeschi & Calhoun, 1996) was administered – a 21-item scale assessing possible positive changes that emerge after traumatic experiences. Items in this self-administered questionnaire were rated on a six-point Likert scale (0 = ‘I did not experience this change as a result of my crisis’, 5 = ‘I experienced this change to a very great degree as a result of my crisis’). Thus, the total score can range from 0 to 105, with higher scores indicating greater posttraumatic growth. Before filling in the PTGI, respondents were asked to answer the following questions as well: ‘Think of the most negative experience/trauma that happened to you in the past 5 years. First, please describe this event and then provide its date’.

When conducting the adaptation process of the instrument into Hungarian, efforts were made to follow the guidelines proposed by Wild et al. (2005). As a first step, three independent professionals (psychologists with master or doctoral levels of education) translated the questionnaire into Hungarian, followed by the development of a consensual version by the same experts. This variant was back-translated into English by an additional independent translator. Tedeschi and Calhoun's team, the authors of the original questionnaire, have checked the back translation and suggested some minor changes that were incorporated in the final Hungarian version (the full text of this adaptation is available as supplemental material to this article on the publisher's website, available at http://dx.doi.org/10.1080/21642850.2014.905208).

### 2.3. Statistical analyses

In order to identify the factor structure that best fits the data, a series of confirmatory factor analyses were performed using maximum-likelihood parameter estimates with the Mplus 7.0 software (Muthén & Muthén, 2012). In addition to the most commonly investigated first- and second-order solutions, we tested the appropriateness of bifactor models as well. This latter kind of model allows for separating the role of the general and domain-specific factors as it – contrary to traditional second-order models – allows all items to load directly onto a general factor (Posttraumatic Growth) as well as on a domain-specific factor (such as ‘Personal Strengths’ or ‘Spiritual Change’). Some recent studies suggest that this measurement structure may be a more effective approach to model construct-relevant multidimensionality (Brunner, Nagy, & Wilhelm, 2012; Reise et al., 2010, 2013) and can contribute to the clarification of inconsistent results on the factor structure of instruments designed to measure complex constructs (Mészáros et al., 2014).

Six models were tested based on accumulated contemporary research. Model 1 was a single factor solution with one general posttraumatic growth factor responsible for all 21-item responses (cf. Anderson & Lopez-Baez, 2008; Joseph et al., 2005; Kovács, Balog, & Preisz, 2012; Sheikh & Marotta, 2005). Model 2 was a three-factor solution representing the three main correlating contributing factors of posttraumatic growth: Self-Perception, Interpersonal Relationships, and Life Philosophy (cf. Anderson & Lopez-Baez, 2008; Joseph et al., 2005; Powell et al., 2003; Weiss & Berger, 2006). Based on the work of the original test developers, Model 3 consisted of five correlated factors contributing to posttraumatic growth: Relating to Others, New Possibilities, Personal Strength, Spiritual Change, and Appreciation of Life (cf. Tedeschi & Calhoun, 1996). Model 4 was a slightly revised form of Model 3 where, in addition to the five first-order factors, a second-order posttraumatic growth factor was also incorporated (cf. Taku et al., 2008).^1^ Model 5 was a bifactor model, including the three components of Model 2 and the general factor. Finally, Model 6 was an additional bifactor model consisting of the five factors from Model 3 and the general factor of posttraumatic growth (Figure 1).

To compare the adequacy of the different models, several fit indices were employed. Satisfactory degree of fit requires the Comparative-Fit Index and the Tucker–Lewis Index to be higher than 0.90 (Hoyle, 1995). The root mean square error of approximation below 0.05 indicates excellent fit, while a value below 0.08 indicates adequate fit (Browne & Cudeck, 1993). The ideal value for the standardized root mean square residual is below 0.08 (Hu & Bentler, 1999). Finally, Bayesian information criteria were also reported, which do not have a clear cut-off; lower values mean better fit (Hooper, Coughlan, & Mullen, 2008).

Internal consistency was evaluated by calculating Cronbach's alpha, omega total, and omega hierarchical coefficients (Zinbarg, Revelle, Yovel, & Li, 2005) for both the whole instrument and its dimensions according to the best fitting model. Omega total estimates the reliability of a latent factor combining the general and specific factor variance while omega hierarchical estimates the reliability of a latent factor with all other latent construct variance removed (Brunner et al., 2012), thus providing useful information on whether scores for a specific factor can be interpreted with confidence or only the total score (general factor score) should be used. Both kinds of omega coefficients were calculated using the Omega software (Watkins, 2013).

The relationship of PTGI scores with demographic characteristics was evaluated using the Mann–Whitney test (sex) and Spearman correlation coefficients (age and educational attainment). Non-parametric methods were used because of the slight deviation (skewness = −0.297, kurtosis = −0.531) of the distribution of PTGI scores from the normal distribution according to the Shapiro–Wilk test (statistic = 0.98, *p* < .001). In the case of the significant Mann–Whitney statistic, effect size was calculated using the following formula: 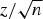.

## 3. Results

Descriptive statistics for the total and subscale scores are also displayed in Table 2. Total PTGI scores were independent of age (*r* = −.055, *p* = .174) and educational level (*r* = .047, *p* = .376), while females in the present sample (*M* = 55.18, SD = 22.45) reported significantly higher PTGI scores (*U* = 22346.5, *p* = .001) than their male counterparts (*M* = 47.13, SD = 23.67). Respondents who completed the survey online (*M* = 58.32, SD = 20.18) also reached higher total scores (*U* = 37855, *p* < .001) than participants answering in paper–pencil format (*M* = 49.19, SD = 24.44). However, the magnitude of the sex and administration mode differences was negligible (effect size *r* of .13 and .18, respectively).

The factor structure of the PTGI was evaluated using confirmatory factor analytic methods. Table 1 presents the fit indices for the six tested models. Our findings suggest that a bifactor model with a 5 + 1 factor structure (Model 6) fits the data best resulting in a significantly better fitting model compared to the other models (see the last column of Table 1). The first bifactor model with a 3 + 1 structure (Model 5) did not have interpretable model fit information due to the emergence of negative variance estimates.

**Table 1.** Model fit indices for competing confirmatory factor analytic models of the PTGI.

	*χ*^2^, *p*	*χ*^2^/df	TLI	CFI	RMSEA (90% CI)	SRSMR	SSA BIC	Difference from Model 6
Model 1 – single factor	1628.8, *p* < .001	8.62	.752	.777	.111 (.106–.116)	.066	45220.7	903.3, *p* < .001
Model 2 – three first-order factors	1211.1, *p* < .001	6.51	.821	.841	.094 (.089–.099)	.055	44812.7	485.6, *p* < .001
Model 3 – five first-order factors	996.8, *p* < .001	5.57	.852	.873	.086 (.081–.091)	.050	44621.2	271.3, *p* < .001
Model 4 – five first-order factors with one second-order factor	102.2, *p* < .001	5.54	.852	.871	.086 (.081–.091)	.052	44628.3	294.7, *p* < .001
Model 6 – bifactor model	725.5, *p* < .001	4.32	.892	.914	.073 (.068–.079)	.044	44385.6	–

Notes: TLI, Tucker–Lewis Index; CFI, Comparative Fit Index; RMSEA, root mean square error of approximation; SRSMR, standardized root mean square residual; SSA BIC, sample-size adjusted Bayesian information criterion.

A detailed analysis (Table 2) of the structure for the best fitting bifactor model revealed that all items loaded significantly onto the global posttraumatic growth factor; however, not all items loaded significantly with their specific domains, suggesting that these items may measure only global posttraumatic growth rather than ‘New Possibilities’ discovered after the traumatic event (Items 11 and 17) or ‘Spiritual Change’ (Items 5 and 18). Since the factor loading of these items on the main factor were quite strong – suggesting that these items represent valuable components of the construct, and the non-significant domain loading is not a result of item inadequacy – we tested a further incomplete bifactor model. This model preserved the structure of Model 6 (Figure 1) with the exception of the domain measuring ‘Spiritual Change’ – the items loaded only onto the global factor in this solution – to represent the applicability of only the total score and the subscale scores of the other four components. However, similar to Models 1–5, this incomplete bifactor model also resulted in a worse model fit compared to the complete bifactor solution of Model 6.

**Table 2.** Descriptive statistics, reliability information, and fully standardized factor loadings from the bifactor confirmatory factor analytic model (Model 6) of the PTGI.

				Standardized factor loading					
Item	*M*	SD	Item-total correlation	Global factor	Relating to Others	New Possibilities	Personal Strength	Spiritual Change	Appreciation of Life
6	2.94	1.59	.509	.484***	.432***				
8	2.40	1.58	.643	.570***	.424***				
9	2.14	1.62	.615	.625***	.302***				
15	2.96	1.59	.598	.682***	.320***				
16	2.75	1.63	.642	.552***	.401***				
20	2.16	1.69	.655	.448***	.403***				
21	2.64	1.57	.565	.643***	.515***				
3	2.45	1.74	.612	.606***		.360***			
7	2.55	1.77	.633	.603***		.502***			
11	2.41	1.68	.724	.637***		.020^NS^			
14	2.20	1.86	.631	.802***		.553***			
17	2.82	1.52	.683	.703***		.067^NS^			
4	2.06	1.77	.621	.675***			.076*		
10	2.91	1.61	.603	.648***			.751***		
12	2.69	1.52	.637	.568***			.121**		
19	2.81	1.72	.593	.609***			.356***		
5	2.08	1.86	.566	.709***				.514^NS^	
18	1.89	1.89	.451	.418***				.596^NS^	
1	3.26	1.58	.505	.605***					.319***
2	2.93	1.66	.560	.608***					.737***
13	2.69	1.66	.654	.499***					.257***
Cronbach's alpha				.931	.862	.858	.805	.699	.752
Common variance				.669	.098	.059	.061	.053	.061
Omega total				.950	.866	.878	.847	.707	.787
Omega hierarchical				.870	.290	.142	.167	.401	.288
Manifest scores [*M* (SD)] for the total PTGI and its subscales				53.75 (22.80)	17.93 (8.31)	12.40 (6.85)	10.46 (5.27)	3.97 (3.28)	8.84 (4.01)

Note: NS, non-significant.

*p < .05.

**p < .01.

***p < .001.

Temporal stability of the Hungarian adaptation was excellent; the test–retest analysis, conducted with a six-week time lag, revealed a very high correlation coefficient (*r* = .90, *p* < .01) between the total scores from the two measurement occasions. The same test–retest reliability coefficients for the subscales were slightly lower and ranged from .71 to .86 (in all cases*, p* < .01).

With regard to internal reliability, the traditional Cronbach's alpha values were very good concerning the total scale and the subscales ‘Relating to Others’, ‘New Possibilities’, and ‘Personal Strength’. In the case of ‘Appreciation of Life’, the alpha value was still good but concerning ‘Spiritual Change’ it was only acceptable (Table 2). The less traditional omega total indicator showed very similar results to those observed using the alpha coefficient: outstanding reliability for the latent general factor and the specific factors ‘Relating to Others’, ‘New Possibilities’, and ‘Personal Strength’. Again, in the case of ‘Appreciation of Life’ omega total was still appropriate, but for ‘Spiritual Change’ reliability was only acceptable. However, coefficient omega hierarchical, estimating reliabilities with the effects of all other factors removed, was considerably low for all specific factors (ranging from .14 to .40, *p* < .01 for all cases) and was high enough only for the general posttraumatic growth factor (Table 2). Data concerning variances showed a very similar pattern: while the general factor accounted for 66.9% of the common variance, the specific factors accounted for only 5.3–9.8% of the common variance (Table 2).

## 4. Discussion

Posttraumatic growth refers to positive psychological changes after highly challenging life crises or traumatic events which – according to previous research evidence over the past years – seems to be a phenomenon existing in several culturally substantially different parts of the world. However, psychometric research on the instrument (PTGI) most widely used to measure this construct revealed substantial differences across samples concerning the factor structure and dimensionality of the PTGI. The aim of this study – in addition to presenting psychometric data on the instrument from another country: Hungary – was to offer an additional factor analytic model to better understand how the components of posttraumatic growth relate to each other and to provide further information regarding the appropriate use of the scale.

Results concerning the comparisons of alternative confirmatory factor analytic models revealed that a bifactor model – including a general posttraumatic growth factor and five specific factors – namely Relating to Others, New Possibilities, Personal Strength, Spiritual Change, and Appreciation of Life – provided the best fit to the present data. If this bifactor solution, never before examined in the literature, is ignored, our results supported a five-factor model of the construct over a single- or a three-factor approach. However, it is worth noting that all three of these previously investigated alternative models clearly showed suboptimal fit for the present sample.

Item analysis of the instrument in the present sample confirmed that each questionnaire item contributed significantly to the measurement of the global posttraumatic growth construct indicating adequate item composition. However, four items (Items 5, 11, 17, and 18) did not load significantly on their designated specific factor (‘New Possibilities’ and ‘Spiritual Change’) indicating that these items were more appropriate indicators of the global posttraumatic growth factor than their specific factors.

Examination of the reliability of the latent PTGI factors revealed that while the general posttraumatic growth factor had strong reliability estimates, omega hierarchical coefficients for the five specific PTGI factors were not high enough to support individual interpretation of these subscales in subsequent research using the PTGI. Results concerning variances also showed that the specific factors did not make a strong contribution to the measurement of posttraumatic growth: the general factor accounted for an overwhelming proportion of total variance in the scores. Therefore, even though the traditional Cronbach's alpha coefficients would allow the separate use of the subdomains, results of the more subtle analyses only support the employment of the total (latent or manifest) PTGI score in future research.

The data of the present study also provided the opportunity to compare the descriptive data for the PTGI in Hungary versus other countries. Our data showed that the total scores reached by our respondents were in the middle of the range as defined by previous research: total PTGI scores found in the present sample were lower when compared to data, for example, from Australia (Morris et al., 2005), Canada (Brunet et al., 2010), or the USA (Sheikh & Marotta, 2005; Tedeschi & Calhoun, 1996), while higher when considering reports from Germany (Maercker & Langner, 2001), the Netherlands (Jaarsma et al., 2006), or Turkey (Dirik & Karanci, 2008), for instance. However, it is important to note that these comparisons can be viewed only as preliminary information on cultural differences since the large variability of the sample characteristics (type of adverse event experienced, time passed since trauma, demographic characteristics, etc.) hinder drawing reliable conclusions on this aspect of the results.

Limitations of the present investigation should also be noted. First, our sample was not representative of the Hungarian population – for instance, male and older individuals were underrepresented in our sample. Thus – even though these demographic variables showed no or very weak association with PTGI scores – the generalizability of our findings is uncertain even for this particular country. Further – although it is a common weakness of research using the PTGI – this study also missed gathering unambiguous information on whether an individual experienced (one or more) trauma(s) as defined by the Diagnostic and Statistical Manual of Mental Disorders in relation to posttraumatic stress disorder (e.g. sexual abuse, serious threat to life) or merely a less decisive although still negative event (e.g. losing an old relative, becoming unemployed). Finally, the electronic and the paper–pencil format of data collection provided different conditions to our subjects which also might have influenced our results. Although it would be possible to investigate the invariance of the factor structures across modes of data collection using multi-group analyses, the number and complexity (particularly that of the bifactor models) of these analyses are beyond the scope of this paper.

We can conclude that our findings support the psychometric adequacy of the Hungarian adaptation of the PTGI measuring posttraumatic growth after ‘psychologically seismic’ events (Tedeschi & Calhoun, 1996; Zoellner & Maercker, 2006). Similar to the conclusion by Osei-Bonsu et al. (2012), our results also indicate that only the total and not the subscale scores of the PTGI should be used in subsequent research using this instrument. Further cross-cultural studies are needed to confirm our findings regarding the appropriate use of total versus subscale scores to assess positive changes in people who experienced loss and trauma. Subsequent studies should also investigate whether the inclusion of further items (for example, in the very short ‘Spiritual Change’ subscale) or the omission of some existing items would contribute to a more stable factor structure and thus more reliable and valid assessment of posttraumatic growth with the PTGI.

## Note

1. A hierarchical (second-order) structure was not tested in case of the three-factor solution (Model 2) because with less than four factors, no statistical difference can be expected between a second-order model and a model with correlated first-order factors (Geiser, 2012).
